# Perioperative varus alignment does not affect short-term patient-reported outcome measures following mobile-bearing unicompartmental knee arthroplasty

**DOI:** 10.1186/s13018-022-02999-5

**Published:** 2022-02-15

**Authors:** Junya Itou, Umito Kuwashima, Masafumi Itoh, Ken Okazaki

**Affiliations:** Department of Orthopaedic Surgery, Tokyo Women’s Medical University, 8-1 Kawada-cho, Shinjuku-ku, Tokyo 162-8666 Japan

**Keywords:** Unicompartmental knee arthroplasty, Patient-reported outcome measures, Oxford partial knee replacement, Forgotten Joint Score-12

## Abstract

**Background:**

Although favorable long-term survival of Oxford unicompartmental knee arthroplasty (UKA) has been reported regardless of postoperative varus alignment, the effect of degree of varus alignment on patient-reported outcome measures (PROMs) remains unclear. Furthermore, the Forgotten Joint Score-12 (FJS-12), which has a low ceiling effect, may be useful for such assessment. The objective of this study was to evaluate short-term clinical outcomes after Oxford UKA in knees with a greater degree of preoperative varus alignment focusing on use of the FJS-12.

**Methods:**

This retrospective study involved 66 knees that had undergone primary Oxford UKA. Based on the hip-knee-ankle angle, the knees were divided into two alignment groups: severe varus group (≥ 185° varus alignment) and a mild varus group (< 185° varus alignment). PROMs, including the FJS-12, Knee Injury and Osteoarthritis Outcome Score, and Knee Society Score, were obtained pre- and postoperatively for assessment of clinical outcomes. In addition, the ceiling effect of the FJS-12 was evaluated.

**Results:**

All PROMs showed significant improvement after surgery. However, there were no statistically significant differences between the severe varus group and the mild varus group. Moreover, no ceiling effect was found for the FJS-12 in this study.

**Conclusion:**

Short-term results were good for Oxford UKA in knees with a greater degree of varus alignment and were not significantly different from those in knees with mild varus alignment.

## Introduction

Unicompartmental knee arthroplasty (UKA) is widely known to have good outcomes in patients with medial knee osteoarthritis or osteonecrosis [1–4]. Oxford mobile-bearing UKA (Oxford UKA; ZimmerBiomet Ltd., Bridgend, UK) is one of the most widely used implants and has been used for a long time [5, 6]. However, total knee arthroplasty (TKA) or high tibial osteotomy (HTO) may be considered for patients with severe varus alignment. Although the survival rate after Oxford UKA is reported to be excellent for varus alignment [7], the effect of the degree of varus alignment on patient-reported outcome measures (PROMs) remains unclear. Kennedy et al. [7] found no relationship between the degree of varus alignment and PROMS using the Oxford Knee Score (OKS). However, the OKS has been reported to have a ceiling effect, and it is unclear whether it is an appropriate measurement [8]. In fact, Kennedy et al. reported a mean of 40 points with a standard deviation of 8 points for the OKS, which has a maximum score of 48 points [7], suggesting that the ceiling effect was present in many patients.

The concept of preserving the constitutional limb alignment in arthroplasty has been attracting attention [9, 10]. Several studies have reported good clinical results using kinematically aligned TKA as a method of preserving constitutional limb alignment [11, 12]. Moreover, using UKA, the patient’s native limb alignment can be restored [13]. Therefore, it is possible that the varus alignment after UKA does not affect PROMs. Nevertheless, patients with a greater degree of varus may have greater concerns about their varus limb alignment preoperatively and be disappointed if there is no change in varus alignment postoperatively. Investigations using more sensitive PROMs with a low ceiling effect are needed.

The Forgotten Joint Score (FJS)-12 is now being widely used as a PROM [14]. This measure has a low ceiling effect and can discriminate results in patients with high scores on other PROMs [15]. There is limited information available on use of the FJS-12 to evaluate outcomes after Oxford UKA in knees with a greater degree of preoperative varus alignment. The objective of this study was to address this gap in the literature, focusing on short-term clinical outcomes.

## Materials and methods

This study had a retrospective design and was approved by our hospital ethics committee (approval number: 4952). Informed consent was obtained via an opt-out procedure.

Seventy-two consecutive knees that were treated by primary Oxford UKA between August 2017 and April 2020 were enrolled. We performed the medial UKA for patients with symptomatic medial compartment disease, no symptoms in other compartments, functional cruciate and collateral ligaments, and preserved range of movement (< 15° extension loss, > 100° flexion). We generally assessed the correctability of limb alignment on a valgus stress radiograph and included patients who showed an anatomical femoro-tibial angle of ≤ 180° on a short film. The exclusion criteria were (1) PROMs including FJS-12 not obtained pre- and postoperatively, (2) lateral UKA, and (3) long-leg standing radiographs not obtained pre- and postoperatively. Finally, the study included 66 knees of 59 patients (13 men, 46 women). Mean age at surgery was 75.3 ± 7.1 years and mean body mass index (calculated as kg/m^2^) was 25.1 ± 4.4. Fifty-four of the 66 knees had primary osteoarthritis and 12 had osteonecrosis.

The knees were divided according to the preoperative and postoperative hip-knee-ankle (HKA) angle measured on digital long-leg standing radiographs into two alignment groups: a severe varus group (≥ 185° of varus alignment) and a mild varus group (< 185° of varus alignment). Postoperative long-leg standing radiographs were obtained at 1 year after surgery. The pre-postoperative change in the radiological parameter (ΔHKA) was calculated.

### Surgical technique

All surgical procedures were performed using the same technique and by any of four knee surgery specialists, all of whom were trained in knee replacement surgery. Oxford UKA was performed using a minimally invasive approach with Microplasty instruments and a tourniquet [16, 17]. The tibial component was cemented in all cases, and the femoral side was either cementless or cemented at the surgeon’s discretion. A postoperative closed-suction drain was placed in some cases. Full weight bearing was permitted immediately after surgery in all patients.

### Outcome measures

Clinical outcomes were assessed using the FJS-12, Knee Injury and Osteoarthritis Outcome Score (KOOS) [18], and Knee Society Score (KSS) [19] obtained preoperatively and 1 year postoperatively. Patients were asked by their attending surgeon to complete these PROMs. For patients who underwent bilateral UKA, PROMs were assessed for each knee.

To evaluate the ceiling effect of the FJS-12 for Oxford UKA, a ceiling score was defined according to a previous study [8]. The minimal clinically important difference (MCID) in the FJS-12 score after UKA has been defined as 12.5 points [20]. Therefore, the ceiling score was defined as ≥ 87.5 points (i.e., greater than or equal to the maximal score of 100 minus the MCID). The ceiling effect was deemed to be reached when > 15% of the responders achieved the ceiling score.

### Complications

Complications occurring up to 1 year postoperatively were retrospectively analyzed using the patients’ medical data.

### Statistical analysis

The differences over time within a group were assessed using paired *t* tests. Differences between the groups were assessed by analysis of variance. Spearman’s rank correlation coefficient was used to assess correlations between preoperative and postoperative varus alignment (HKA) and each of the PROMs postoperatively. All statistical analyses were performed using JMP software (SAS Institute Inc., Cary, NC). A *p* value of ≤ 0.05 was accepted as statistically significant.

## Results

### Assessment of perioperative lower limb alignment

Mean HKA was 185.7° ± 4.1° preoperatively and 183.5° ± 3.4° postoperatively. Postoperative ΔHKA was 2.1° ± 3.1° (Table 1).

**Table 1:** Demographics for 59 patients and clinical characteristics in 66 knees

Mean age	75.3 ± 7.1 years
Sex	13 men, 46 women
BMI (kg/m^2^)	25.1 ± 4.4
Diagnosis	54 osteoarthritis, 12 osteonecrosis
Pre-HKA angle	185.7° ± 4.1° (176.9–195.5)
Post-HKA angle	183.5° ± 3.4° (175–191.8)
ΔHKA	2.1° ± 3.1° (− 3.4, 11.7)

BMI, body mass index; HKA, hip-knee-ankle

The 66 knees were divided according to the degree of preoperative HKA (classification 1) into a severe varus group (*n* = 36) and a mild varus group (*n* = 30). Mean HKA was 188.5° ± 2.9° preoperatively and 185.1° ± 3.3° postoperatively in the severe varus group and 182.2° ± 2.0° and 181.7° ± 2.3°, respectively, in the mild varus group (Table 2). There was a significant change in alignment, with mean ΔHKA of 3.4° ± 3.5° in the severe varus group and 0.5° ± 1.4° in the mild varus group (*p* < 0.0001, Table 2).

**Table 2:** Change in HKA angle

	Pre-HKA angle	Post-HKA angle	ΔHKA
*Classification 1 (preoperative)*			
Severe varus group	188.5° ± 2.9°	185.1° ± 3.3°	3.4° ± 3.5°
Mild varus group	182.2° ± 2.0°	181.7° ± 2.3°	0.5° ± 1.4°
*p* value	< 0.0001	< 0.0001	< 0.0001
*Classification 2 (postoperative)*			
Severe varus group	189.0° ± 3.6°	187.4° ± 2.0°	1.5° ± 3.5°
Mild varus group	184.3° ± 3.4°	182.0° ± 2.3°	2.3° ± 2.9°
*p* value	< 0.0001	< 0.0001	0.34

HKA, hip-knee-ankle; Post, postoperative; Pre, preoperative

The 66 knees were similarly divided according to the degree of postoperative HKA (classification 2) into a severe varus group (*n* = 19) and a mild varus group (*n* = 47). Mean HKA was 189.0° ± 3.6° preoperatively and 187.4° ± 2.0° postoperatively in the severe varus group and 184.3° ± 3.4° and 182.0° ± 2.3°, respectively, in the mild varus group (Table 2). There was no significant change in alignment, with mean ΔHKA of 1.5° ± 3.5° in the severe varus group and 2.3° ± 2.9° in the mild varus group (*p* = 0.34, Table 2).

### Assessment of perioperative PROMs

All PROMs showed significant improvement postoperatively (Table 3). However, there were no statistically significant differences in PROMs between the severe and mild varus groups either preoperatively or postoperatively according to whether we used classification 1 (preoperative varus; Tables 4 and 5) or classification 2 (postoperative varus; Tables 6 and 7). Moreover, there was no statistically significant difference in the change in KSS for satisfaction between the two groups (Table 8).

**Table 3:** change in each PROM after surgery

	FJS-12	KSS total	KOOS (pain)	KOOS (symptoms)	KOOS (ADL)	KOOS (sports)	KOOS (QoL)
Before surgery	15.3 ± 13.1	84.1 ± 27.1	46.5 ± 19.2	58.1 ± 22.8	56.6 ± 18.3	23.3 ± 19.3	28.0 ± 17.3
After surgery	48.4 ± 26.0	130.6 ± 26.5	80.9 ± 16.7	82.9 ± 13.9	83.1 ± 14.9	52.1 ± 24.2	63.8 ± 25.3
*p* value	< 0.0001	< 0.0001	< 0.0001	< 0.0001	< 0.0001	< 0.0001	< 0.0001

ADL, activities of daily living; FJS-12, Forgotten Joint Score; KOOS, Knee Injury and Osteoarthritis Outcome Score; KSS, Knee Society Score; PROM, patient-reported outcome measure; QoL, quality of life

**Table 4:** Comparison of preoperative value for each PROM between the severe varus group and the mild varus group (classification 1; preoperative varus)

Before surgery	FJS-12	KSS total	KOOS (pain)	KOOS (symptoms)	KOOS (ADL)	KOOS (sports)	KOOS (QoL)
Severe varus	13.7 ± 12.3	81.1 ± 24.2	46.7 ± 18.0	58.0 ± 22.2	57.0 ± 15.7	22.9 ± 17.7	29.6 ± 14.3
Mild varus	17.1 ± 14.0	87.6 ± 30.3	46.2 ± 20.8	58.2 ± 23.9	56.1 ± 21.2	23.8 ± 21.4	26.0 ± 20.5
*p* value	0.30	0.34	0.91	0.97	0.83	0.84	0.40

ADL, activities of daily living; FJS-12, Forgotten Joint Score; KOOS, Knee Injury and Osteoarthritis Outcome Score; KSS, Knee Society Score; PROM, patient-reported outcome measure; QoL, quality of life

**Table 5:** Comparison of postoperative value for each PROM between the severe varus group and the mild varus group (classification 1, preoperative varus)

After surgery	FJS-12	KSS total	KOOS (pain)	KOOS (symptoms)	KOOS (ADL)	KOOS (sports)	KOOS (QoL)
Severe varus	48.7 ± 21.5	134.7 ± 19.1	82.4 ± 13.4	82.5 ± 12.0	85.4 ± 11.7	51.8 ± 23.1	65.4 ± 21.9
Mild varus	48.0 ± 30.9	125.8 ± 33.0	79.1 ± 20.0	83.4 ± 16.1	80.2 ± 17.8	52.6 ± 25.8	61.8 ± 29.1
*p* value	0.17	0.90	0.43	0.79	0.15	0.88	0.57

ADL, activities of daily living; FJS-12, Forgotten Joint Score; KOOS, Knee Injury and Osteoarthritis Outcome Score; KSS, Knee Society Score; PROM, patient-reported outcome measure; QoL, quality of life

**Table 6:** Comparison of preoperative value for each PROM between the severe varus group and the mild varus group (classification 2, postoperative varus)

Pre-Op	FJS-12	KSS total	KOOS (pain)	KOOS (symptoms)	KOOS (ADL)	KOOS (sports)	KOOS (QoL)
Severe varus	15.5 ± 14.9	81.7 ± 26.1	50.8 ± 16.1	65.6 ± 18.9	59.4 ± 16.2	21.3 ± 19.7	31.9 ± 14.7
Mild varus	15.2 ± 12.5	85.0 ± 27.8	44.8 ± 20.2	55.0 ± 23.8	55.5 ± 19.1	24.1 ± 19.3	26.4 ± 18.2
*p* value	0.91	0.65	0.24	0.09	0.43	0.59	0.25

ADL, activities of daily living; FJS-12, Forgotten Joint Score; KOOS, Knee Injury and Osteoarthritis Outcome Score; KSS, Knee Society Score; PROM, patient-reported outcome measure; QoL, quality of life

**Table 7:** Comparison of postoperative value for each PROM between the severe varus group and the mild varus group (classification 2, postoperative varus)

After surgery	FJS-12	KSS total	KOOS (pain)	KOOS (symptoms)	KOOS (ADL)	KOOS (sports)	KOOS (QoL)
Severe varus	52.0 ± 24.1	137.6 ± 18.1	85.0 ± 11.2	84.0 ± 12.3	87.4 ± 9.8	54.4 ± 23.9	71.0 ± 22.4
Mild varus	46.9 ± 26.8	127.8 ± 28.9	79.2 ± 18.2	82.5 ± 14.6	81.3 ± 16.2	51.2 ± 24.5	60.9 ± 26.1
*p* value	0.47	0.17	0.19	0.69	0.13	0.63	0.14

ADL, activities of daily living; FJS-12, Forgotten Joint Score; KOOS, Knee Injury and Osteoarthritis Outcome Score; KSS, Knee Society Score; PROM, patient-reported outcome measure; QoL, quality of life

**Table 8:** Change in the satisfaction value in the KSS

	Pre-KSS satisfaction	Post-KSS satisfaction	ΔKSS satisfaction
*Classification 1 (preoperative)*			
Severe varus group	13.1 ± 7.3	28.1 ± 7.2	15.0 ± 9.1
Mild varus group	13.1 ± 7.1	26.7 ± 9.4	13.6 ± 10.9
*p* value	0.96	0.52	0.57
*Classification 2 (postoperative)*			
Severe varus group	13.7 ± 7.8	29.1 ± 7.5	15.2 ± 9.5
Mild varus group	12.8 ± 6.9	26.8 ± 8.5	14.0 ± 10.2
*p* value	0.61	0.32	0.64

KSS, Knee Society Score

**Fig. 1:**
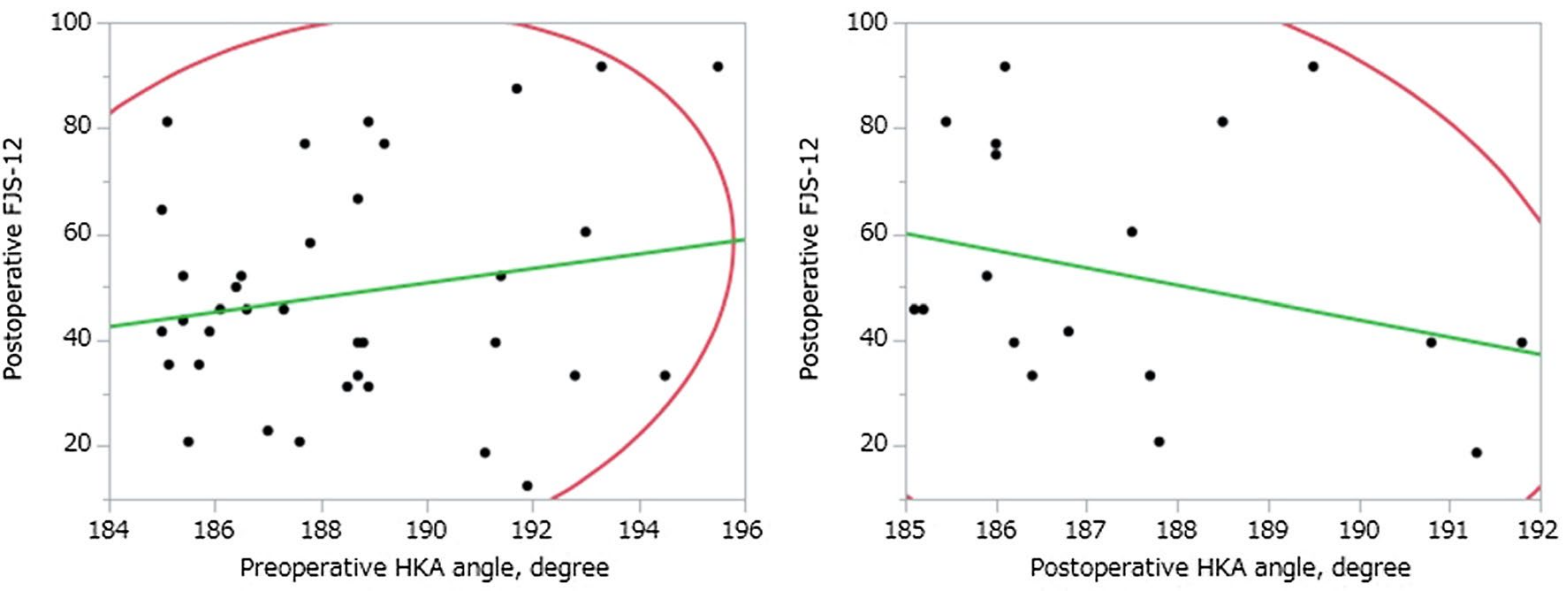
Preoperative degree of varus alignment (HKA) compared with the postoperative FJS-12 value. There was no significant correlation between preoperative varus alignment (HKA angle) and the postoperative FJS-12 value (*r* = 0.18, *p* = 0.27) or between postoperative varus alignment (HKA angle) and the postoperative FJS-12 value (*r* = 0.27, *p* = 0.24). HKA, hip-knee-ankle; FJS-12, Forgotten Joint Score

**Fig. 2:**
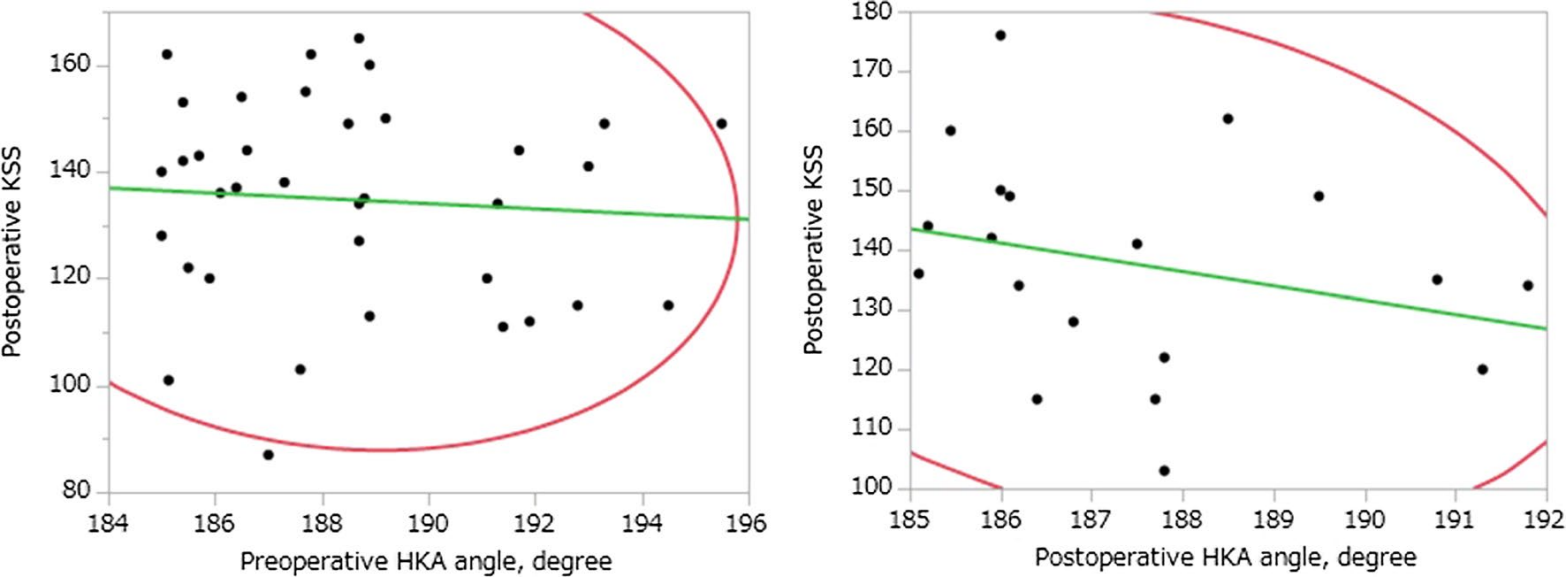
Preoperative degree of varus alignment (HKA) versus postoperative KSS. There was no significant correlation between preoperative varus alignment (HKA angle) and postoperative KSS (*r* = 0.07, *p* = 0.66) or between preoperative varus alignment (HKA angle) and postoperative KSS (*r* = 0.27, *p* = 0.26). HKA, hip-knee-ankle; KSS, Knee Society Score

The correlations of postoperative PROMs with the preoperative and postoperative HKA angle were assessed in the severe varus group. The FJS-12 value was not significantly correlated with either the preoperative or postoperative HKA angle (preoperative varus: *r* = 0.18, *p* = 0.27; postoperative varus: *r* = 0.27, *p* = 0.24; Fig. 1). Similarly, postoperative KSS showed no significant correlation with either the preoperative or postoperative HKA angle (preoperative varus: *r* = 0.07, *p* = 0.66; postoperative varus: *r* = 0.27, *p* = 0.26; Fig. 2). Furthermore, there was no significant correlation between any of the postoperative KOOS subscale values and preoperative or postoperative varus alignment (HKA).

**Fig. 3:**
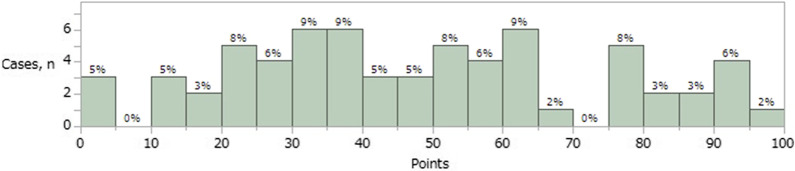
Histogram showing postoperative FJS-12 values. Less than 15% of participants achieved the ceiling score (defined as 87.5 points or more). There was no ceiling effect of FJS-12 following UKA. FJS-12, Forgotten Joint Score; UKA, unicompartmental knee arthroplasty

The ceiling effect of the FJS-12 was assessed using a histogram (Fig. 3). This showed that less than 15% of participants achieved the ceiling score, defined as 87.5 points or more, indicating that there was no ceiling effect for FJS-12 following UKA.

### Complications

There were no revision cases requiring conversion to TKA. Furthermore, no additional surgery following UKA was required during the study period. There were no cases of bearing dislocation or fatal thromboembolism.

## Discussion

The most important finding of this study was that the short-term results of Oxford UKA for knees with a greater degree of preoperative and postoperative varus alignment were good. There were no significant differences in the results for any of the PROMs used, including the FJS-12, according to whether varus alignment (HKA) was classified as ≥ 5° or < 5° either preoperatively (classification 1) or postoperatively (classification 2). Furthermore, there was no correlation of any of the PROMs with either preoperative HKA in the varus group based on classification 1 or postoperative HKA in the varus group based on classification 2. Moreover, the FJS-12 had no ceiling effect in this study.

Candidates for UKA have recently expanded to include younger and more active patients [21]. UKA is often compared with HTO [22–24], and the indications for surgery may also overlap. Although patients with severe varus alignment (HKA ≥ 185°) may be candidates for HTO, this study demonstrates that PROMs following Oxford UKA for knees with more than 5° of varus alignment were relatively good and not significantly different from those in the mild varus group. A study by Jin et al. that included a propensity score matching analysis found that the clinical outcomes were better after UKA than after HTO [25]. Moreover, Kennedy et al. [7] found no correlation between the degree of postoperative varus alignment and postoperative PROMs, which is in line with our present findings. The results of our study support the concept of the Oxford UKA technique [16], which aims to achieve correct ligament balance and restore constitutional limb alignment.

The definition of ceiling effect has been controversial [8]. Various methods have been reported, with some authors using the maximum score and others using scores within 1 standard deviation of the highest score [15, 26]. In this study, the ceiling effect was rigorously evaluated using the MCID according to the method described in a previous study [8]. The MCID for the FJS-12 has been reported to be 12.5 points [20]. Therefore, for example, an FJS-12 of 90 points may not show a clinically significant difference from a maximum FJS-12 score of 100 points. Using this definition, we determined that there is no ceiling effect for the FJS-12 following UKA.

When classification 1 was used, the ΔHKA in the severe varus group was significantly greater than that in the mild varus group. This suggests that the severe varus group in classification 1 included many cases with significant intra-articular deformities as a result of cartilage and bone wear and that the native medial joint line was restored by relatively thin bone resection and/or insertion of a relatively thick bearing [27]. Kuwashima et al. reported that correction of limb alignment was correlated with restoration of medial joint height in fixed bearing UKA [28]. Using classification 1, significant correction of limb alignment was achieved in varus cases. Nevertheless, in some cases with preoperative varus, correction of limb alignment was inadequate as a result of extraarticular deformity, and these cases were subsequently classified as having postoperative varus according to classification 2. Severe preoperative varus alignment has been reported to affect postoperative alignment following UKA [22]. However, in our mild varus group, there was very little change in alignment postoperatively. Given the minimal effect of osteophytes and intra-articular deformities, the concept of resurfacing surgery in Oxford UKA could have been directly implemented [13].

This study has several limitations. First, it did not include a postoperative assessment of radiographic parameters, such as radiolucent lines or malposition of the implant. The correlation between the common finding of physiological radiolucent lines following Oxford UKA and PROMs is still unclear [29]. Although most radiolucent lines are considered not to progress, some adverse phenomena such as micromotion of the implant may be involved [22]. In addition, poorer outcomes have been observed with malpositioning of the implant [30]. However, no apparent implant failure was observed in this cohort. Second, the results were assessed only in the short term and may have changed over time. A previous study found that scores for clinical outcomes, including the OKS, were highest at 1 year postoperatively and declined over a 10-year period [6]. In terms of evaluating the efficiency of the FJS-12, the assessment at 1 year postoperatively may be considered appropriate. Third, the sample size was relatively small. Further studies in larger cohorts are warranted. Fourth, there were a few cases of marked malalignment, such as a fixed varus deformity > 15°, which is not indicated for Oxford UKA [4, 16]. Although we assessed the correctability of preoperative varus deformity based on a valgus stress radiograph, a few patients had > 10° varus postoperatively. The results for marked malalignment remain unclear.

## Conclusions

Short-term results for Oxford UKA were good, with no significant difference in outcome according to the degree of varus alignment. The FJS-12 had no ceiling effect when used to assess PROMs following Oxford UKA and was a useful outcome measure.

## Data Availability

The datasets used and/or analyzed during the present study are available from the corresponding author on reasonable request.

## Abbreviations

FJS-12: Forgotten Joint Score-12
HKA: Hip-knee-ankle
HTO: High tibial osteotomy
KOOS: Knee Injury and Osteoarthritis Outcome Score
KSS: Knee Society Score
MCID: Minimum clinically important difference
OKS: Oxford Knee Score
Oxford UKA: Oxford mobile-bearing unicompartmental knee arthroplasty
PROMs: Patient-reported outcome measures
TKA: Total knee arthroplasty
UKA: Unicompartmental knee arthroplasty
